# Left atrial scar identification and quantification in sinus rhythm and atrial fibrillation

**DOI:** 10.1002/joa3.12421

**Published:** 2020-09-02

**Authors:** James Mannion, Joseph Galvin, Usama Boles

**Affiliations:** ^1^ Cardiology Department, Heart and Vascular Centre Mater Private Hospital Dublin Ireland

**Keywords:** atrial cardiac remodeling, atrial fibrillation, catheter ablation, electrophysiology, pulmonary veins

## Abstract

Identification and quantification of low voltage areas (LVA) in atrial fibrillation (AF), identified by their bipolar voltages (BiV) via electro‐anatomical voltage mapping is an area of interest to prognosis of AF free burden. LVAs have been linked to diseased left atrial (LA) tissue which results in pro‐fibrillatory potentials. These LVAs are dominantly found within the pulmonary veins, however, as the disease progresses other areas of the LA show low voltage. The scar burden of the LA is linked to recurrence of the arrhythmia and can be a target of further modification. This burden is classically assessed once sinus rhythm (SR) is attained, but this is susceptible to operator variability with overestimated dense LA scar (<0.2 mV) and underestimated diseased LA tissue (<0.5 mV). The novel automated voltage histogram analysis (VHA) tool may increase accuracy, however, is yet to be fully validated. A recent study indicates that LVAs can be assessed just as reliably in AF as SR, but BiV is lower with linear correlation to SR values (0.24‐0.5 mV respectively). In this paper, we review current data as well as review current methods of identifying, quantifying, and grading LA scar. We also compared AF vs SR voltages of a patient undergoing catheter ablation in our site using our VHA tool to compare the results. In keeping with the cited papers, we found lower voltages in our patient measured in AF. This area warrants further study to assess correlation in more patients, with view to developing prognostic and therapeutic grading systems.

## Graphical abstract

Review article discussing the invasive and noninvasive techniques to identify and quantify left atrial scar using myocardial voltages. We discuss the novel voltage histogram analysis tool which may be used during electroanatomical voltage mapping to compare voltages in sinus rhythm and atrial fibrillation.

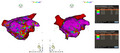

## 1 BACKGROUND

The lifetime risk of atrial fibrillation (AF) can be as high as 23.8% in men and 22.2% in women.^1^ It is well‐established that there are many risk factors for AF through a variety of mechanisms. Some modifiable risk factors include hypertension, obesity, thyroid dysfunction, obstructive sleep apnea, excess alcohol, or caffeine. Nonmodifiable risk factors include age, male gender, and established structural heart disease.^2^, ^3^ One of the more interesting risk factors for AF, however, is the duration of persistent AF. “AF begets AF”^4^ and increasing scar burden.^5^

## 2 PATHOPHYSIOLOGY OF MYOCARDIAL REMODELING IN AF

Myocardial tissue which remains in AF for 24 hours or more has evidence of ion channel and electrophysiological remodeling, this remodeled tissue or scarring supports ongoing re‐entry and frequency of fibrillatory triggers in other areas of the LA. These triggers and re‐entry pathways promote further intrinsic structural change serving sustained longer durations of AF.^4^, ^5^, ^6^ Atrial remodeling, through multiple mechanisms, can enhance the number of ectopic beats that initiate re‐entrant circuits^6^ in addition to the main source of pulmonary vein potentials.^7^ One of the pathophysiological theories, for example, is that sustained rapid atrial depolarization decreases inward L‐type calcium currents and increases outward potassium ones.^6^ In addition, there is structural remodeling of the atrium. This is a process of myocyte injury and fibrosis. One such cause is from prolonged exposure to risk factors which promote fibrogenesis, such as hypertension, diabetes, or congestive cardiac failure.^8^, ^9^ Myocyte loss and fibrosis results in a reduction of ion channels and fibers which are integral to contractility. Atrial dilation is a late feature of this fibrotic process, and greater accommodation of re‐entry circuits is possible through progressively increased atrial size.^10^

## 3 PULMONARY VEIN POTENTIALS AND ANTRAL SCAR

Pulmonary vein “PV” Ostia are the sources of ectopy and scar related substrates that initiate and promote AF.^8^ The diameter of the PVs may play a pivotal role.^11^ Hence, circumferential isolation of all PVs, has established itself as a mainstay of treatment for AF. Pulmonary vein isolation (PVI) is superior than standard anti‐arrhythmic therapy owing to that, PVs accommodate pacing cells, transitional cells, and purkinje cells.^12^, ^13^, ^14^, ^15^ Additionally, cardiomyocytes found in the PV have subtle ion channel and depolarization potential that put them at increased risk for initiating and sustaining arrhythmia.^4^ Wide area circumferential ablation of PVs is often not enough for rhythm maintenance. Further ablation substrate is usually located and targeted via low voltage guidance and 3D mapping.^16^ However, another study contradicted that suggesting high voltage areas would be a valid target to isolate the PVs.^17^

Low voltage areas (LVA) on electroanatomic mapping correlate with areas of myocardial scarring found on MRI with late gadolinium enhancement.^18^ In a sentinel study conducted by Yagishita Atsuhko et al,^19^ they identified low bipolar voltage (BiV) areas on a 3‐D electroanatomic system in both sinus rhythm (SR) and AF, in order to map and compare these areas of scarring in both rhythms. This study had two conclusions, firstly there was nearly identical characterization of LA regions exhibiting low voltage on electroanatomical voltage mapping (EAVM) irrespective of the rhythm. The cut‐off voltage values to identify these areas of scarring must be increased, but appear to be equally as reliable in AF as in SR. The voltage values of scarring in AF and SR correspond in a linear fashion. Secondly it was found that in AF patients, the PV antra exhibited lower voltages than other LA regions, whereas no voltage differences were observed in control patients. This may suggest that early structural changes commonly involve the PV antra initially before affecting the LA body.^19^

This is significant as in a substantial prospective study of a cohort containing paroxysmal and persistent AF who underwent catheter ablation, atrial scarring (identified by DE‐MRI) was a worrying predictor of reverting back to AF post procedurally. This risk was proportional to the extent of scarring.^20^ A recent study by Solimene et al used a strict ablation protocol and ablation index (AI) thresholds in relation to contiguity and quality of lesion can drastically reduce PVI variability despite variable practitioner skill levels and different catheters utilized.^21^

## 4 NONINVASIVE MODALITIES OF ASSESSING SCAR BURDEN

Scarring can be assessed by a few modalities. Firstly, noninvasive cardiac MRI can be used with delayed gadolinium enhancement. A common technique to localize areas of scarring is the image intensity ratio (IIR), which normalizes mean myocardial image intensity in each sector, and this has been shown to accurately assess the extent to which the LA has fibrosed, in SR or relatively rate controlled AF.^20^, ^21^ Marrouche et al in a multicenter trial has investigated variable local guidelines for late contrast injection MRI. The exact numbers of patients with MRI conducted in SR vs AF was unspecified.^20^ In another study, however, by Zghaib et al 7 of 26 patients presented to their LGE‐MRI in AF which required direct current cardioversion (DCCV). Their entire cohort was assessed in SR.^21^ This study found good correlation between BiV, point‐by‐point mapping and late gadolinium enhanced MRI using IIR.^22^ The Utah classification can be used with cardiac MRI to quantify the degree of LA fibrosis,^23^ in their study over 90% of patients had images attained in SR.^24^ Utah I ≤ 5%, II > 5%, III > 20% ≤35%, and IV > 35%. This system has clinical significance. DE‐MRI established a key role and became the gold standard for LA fibrosis identification and classification.^24^ The Utah classification of scarring was an independent risk factor with recurrence of AF after PVI.^20^, ^23^, ^25^ Poor spatial resolution in the myocardium of the LA means that DE‐MRI is both challenging to accurately perform and also requires specialist interpretation. It is limited thus in its availability.^24^

## 5 INVASIVE MODALITIES OF ASSESSING SCAR BURDEN

Secondly, we have EAVM of the LA. This is done via commonly available mapping systems. In one study conducted by Herczeg et al, the myocardium is mapped according to it's BiV area using CARTO 3D, Biosense Webster Inc, USA. mapping system. It was found that these areas of low voltage exhibit re‐entry circuitry and triggers, the cornerstones of AF pathophysiology, during the arrhythmia when measured.^24^ Herczeg et al used a cut‐off <0.5 mV was to identify areas of low voltage.^26^ These LVA correlate with diseased myocardium/AF substrate. These measurements are conventionally attained when the patient is in SR, however, a study from Yagishita et al have shown that these areas of fibrosis follow a linear distribution of voltage when also attained in AF, but perhaps just requiring a different cut‐off range and different voltage criteria. These voltages would generally be lower.^19^

## 6 ANATOMICAL DISTRIBUTION AND METHODS OF THE SCAR QUANTIFICATION

Yagishita has shown that EAVM of scarred tissue in AF has similar outcomes to that in SR when the voltage criteria are adjusted. As we have discussed, the ostia of the pulmonary veins are recognized as the first area of remodeling, but Yagishita also comments on the fact that they found some of the lowest regional voltages in the septal wall.^19^ Over 95% of values of the control cohort attained in this area were greater than 1.17 mV, and concludes that perhaps a definition of <1.17 mV during SR could be utilized as a threshold to recognize early stages of scar formation in the left atrium.^19^

However, Dublin group in 2019, subcategorized these voltages even more, with interesting outcomes. In their ablations, patients were measured and ablated in SR, or when paced at 600 ms CL via the coronary sinus. They used a classical wide area circumferential ablation and a circular multipolar catheter containing 20 electrodes for EAVM, also using an ablation catheter to add extra data as needed. Left atrial (LA) appendage, pulmonary vein, and mitral annulus data were manually excluded. Their voltages of ≤0.2 mV were classified as “Dense LA Scar” and points ≤0.5 mV were designated “Diseased LA Tissue”^24^, these definitions were each subdivided into quartiles based on the percentage of LA area covered by disease, this generated classes I to IV of disease for both categories. They used an automated offline programme to assess the myocardium and designate scores with great accuracy and reproducibility‐ CARTO3 Voltage Histogram Analysis (VHA). These classes were <1%, 1%‐3%, 3.1%‐8% and finally >8% for dense scar tissue (≤0.2 mV). The corresponding ranges for “Diseased LA Tissue” were <9%, 9%‐18%, 18.1%‐31% and >31% for the final class.

It is important to identify this scar burden. Rolf et al have shown in addition to standard WACA/ PVI, that ablation of LVAs when identified via EAVM have better results at 1 year.^16^ The identification and analysis of this scar tissue to date has faced obstacles such as lack of availability of MRI and no consistent reproducible method of quantification via BiV.

## 7 IDENTIFICATION OF THE SCAR BURDEN IN AF AND IN SR

Multiple factors are known to influence voltage results. Wider or narrower electrode spacing distances influences travel time (or along the same lines, lesser or greater velocity of the depolarization respectively) and will result in differences in amplitude and thus morphology of recording.^27^

There is also evidence to show that increasing interelectrode distance leads to increased voltages but only in select patients.^28^ Furthermore, larger sized electrodes which cover greater area may potentially demonstrate increased voltage readings, such as those elicited by Marcus et al^29^ although this is not the case universally. Readings can depend on the underlying myocardial properties. In the presence of atrial scar tissue, the voltage from both normal and diseased myocardial tissue can be grouped by larger electrodes and result in reduced voltage readings, as reported by Anter et al”^30^ In contrast, when utilizing catheters with reduced electrode size it has been demonstrated with statistical significance to yield increased voltage amplitudes.”^31^

The catheter contact force to underlying myocardium also influences the recorded voltage, but only to a point. This has been demonstrated as low contact forces (below 0.05 Newtons), where there is a demonstratable positive relationship between increasing the force and the voltages recorded.^32^

Finally, increasingly dilated remodeled atria or those acutely dilated under strain are associated with reduced mean atrial voltages.^33^, ^34^ However, these variables may largely be negated when using the same operator with a standard practice. In our example we have used the same patient, operator, catheter, and number of contact points to minimize these effects.

Yagishita et al showed that scar burden in AF vs SR is comparable in EAVM when thresholds are altered to lower levels for AF. LVA cut‐off measurement of <0.5 mV in AF is comparable to <1.5 mV in SR. This was true for both native and induced AF.^19^ Neither mapping time nor LA volume showed any significant difference between SR and AF in this study.^19^ Yagishita split the LA into nine regions for comparison (roof, posterior, inferior, anterior, septal, lateral, LAA, RPVa, LPVa), and there was no distributional difference of LVAs between these regions in SR and AF.^19^

There was linear voltage correlation between the two rhythms with generally higher voltage in SR than in AF throughout all regions. The highest voltages were found within the left atrial appendage (LAA) followed by the lateral wall. This was true for both AF and SR, as well as paroxysmal and nonparoxysmal AF. LA BiV showed higher values in patients with pAF than non‐pAF. This was regardless of rhythm at time of measurement.^19^

Herczeg et al conducted their study of the novel automated voltage analysis tool with patients in SR. They found generally lower voltages in patients with more persistent AF over paroxysmal,^26^ similar to recent findings by Rodríguez‐Mañero et al^35^

Oakes examined atrial scarring in MRI with over 90% of their patients being in SR at the time of MRI.^24^ There was no direct imaging comparison of patient fibrosis between AF and SR in this study. Patients in fast AF were a recognized limiting factor in this study as it made obtaining values more difficult.^24^

It is established that patients with more persistent clinical AF demonstrate lower voltage areas on MRI and BiV. As such we expect more extensive scar tissue to have established itself throughout the LA. We have seen that similar linear scar tissue identification results can be obtained in both SR and AF when the voltage criteria have been adjusted, but further research is required on the comparability of AF to SR throughout the imaging modalities.

## 8 EXAMPLE OF AUTOMATED VOLTAGE HISTOGRAM ANALYSIS IN AF AND IN SR

The VHA tool is an offline software created by Biosense‐Webster which has been studied in the analysis of fast anatomical mapping (FAM).^26^, ^36^ As with our standardized PVI procedure, the catheter we have used is a 20 pole LASSO D‐curve, 7 French with 4.5‐millimetre pairs spacing (Biosense‐Webster, J & J Medical NV/SA, Belgium). The intrinsic voltage of the atrium is analysed (via the color code) where the color accredited depends on the value of voltages (ie at 0.1 mV, 0.2 mV …etc). Then a table of the values is produced which allows for accurate analysis and quantification of total area of each voltage range, which minimizes operator variability. The tool calculates the total area of the preselected atrium which falls under each voltage color code and summarizes this information into an area table. This table can be used to calculate the proportion of the myocardium falling under the categories of normal tissue, diseased or dense scar. To maximize accuracy and minimize interference, areas such as the mitral annulus, the trans‐septal puncture, the LAA, and the pulmonary veins are manually removed from the analysis by a trained technician.

**FIGURE 1:**
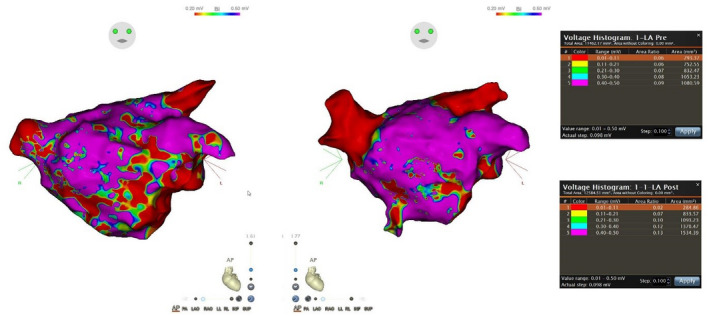
Voltage Histogram Analysis tool tables. The left image represents mapping in atrial fibrillation preablation and predirect current cardioversion (DCCV), this image is coupled with the upper table entitled “LA Pre”. This demonstrates a greater combined area in the aliquots <0.2 mV than the second image, which was mapped in sinus rhythm (SR), post‐DCCV, and ablation. The second map is coupled with the bottom table entitled “LA Post” which demonstrates far more area in the higher voltage aliquots. As shown in the maps and tables each voltage range is color‐coded: A myocardial reading of 0‐0.1 mV = Red; 0.11‐0.2 mV = Yellow; 0.21‐0.3 mV = Green; 0.3‐0.4 mV = Teal; and finally, 0.4 mV and above = Purple

**FIGURE 2:**
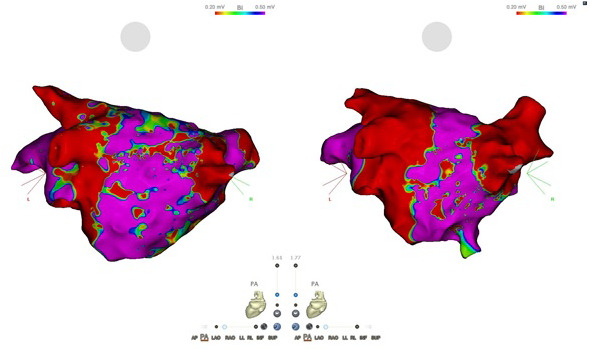
Postero‐Anterior (PA). The left image shows left atrial (LA) bipolar electroanatomical voltage mapping, preablation in atrial fibrillation. The right image shows the LA in sinus rhythm postablation and postdirect current cardioversion. Such as in Figure 1, a myocardial reading of 0‐0.1 mV = Red; 0.11‐0.2 mV = Yellow; 0.21‐0.3 mV = Green; 0.3‐0.4 mV = Teal; and finally, 0.4 mV and above = Purple

For the purposes of our demonstration, the VHA was set to values of about 0.1 mV aliquots. Each aliquot was then represented by a different color and was given an area in mm^2^. This table and our FAM are demonstrated in Figure 1 with an alternative view demonstrated in Figure 2.

As shown in (Figures 1 and 2) via color coding, diffuse LVAs are more evident in AF prior to ablation with red and yellow representing voltages of <0.2 mV. Table 1 demonstrates this proportional difference in a summary. This patient was ablated in AF with WACA extending around both right PVs and left PVs. The patient was cardioverted to SR and reassessed. Prior to our VHA analysis, we removed the PVs and area within utilized ablation lines, the mitral annulus, LAA, and trans‐septal puncture site.

**TABLE 1:** Compares low voltage areas and dense scar distribution in electroanatomical voltage mapping between atrial fibrillation and sinus rhythm as per our voltage histogram analysis tool. A voltage reading of <0.5 mV recognized as “Diseased LA Tissue” and <0.2 mV recognized as “Dense LA Scar”

Variables	AVM in AF	AVM in SR
Total area <0.5 mV (%)	43.3	35.8
Total area <0.5 and >0.2 mV (%)	34.18	23.57
Total area <0.2 mV (%)	9.15	12.28

## 9 ANATOMICAL VARIATION OF SCAR DISTRIBUTION AND CLINICAL SIGNIFICANCE OF DIFFERENCES

A recent study by Benito et al looked at trends of fibrotic changes in AF using LGE‐ MRI has found that areas of scar are often located on the posterior wall and around the antrum of the left pulmonary vein.^37^ These findings correlate somewhat to our patient with persistent AF, demonstrating low voltage posteroinferiorly. While in AF prior to intervention and DCCV, our VHA has demonstrated most of the voltages <0.2 mV were located on the antero‐septal wall as shown.

A previous study has shown correlation between visual assessment and our VHA tool, however, visual assessment of the burden of dense scar can be overestimated, and moderate scar can be underestimated in comparison.^36^ Conversely, VHA analyses and identification of disease/fibrillatory potentials in AF may also be overestimated because of lower but linear voltages in AF. It has been suggested that LVAs of 0.24 mV in AF correspond to 0.5 mV in SR.^35^ The target for further non‐PV substrates might be more accurately identified now in AF with adjustment of voltage criteria. Further data are required in the area as another study conflicted this information citing that there were large discrepancies between low voltage locations between SR and AF.^38^

The optimal electrophysiological target used to identify substrate perpetuating AF has evolved dramatically over the last several years. The initial STAR AF trial^39^ showed that in high burden/persistent AF, PVI with concurrent complex fractionated electrogram (CFE) targeted ablation had much greater freedom from AF at 1 year (74%) than PVI (48%) or CFE (29%) alone. Conversely in the STAR AF II study^40^ there was no difference between treatment arms of PVI + linear ablation or PVI + CFE vs PVI alone (*P* value = .15). The authors were unable to identify a cause of this finding and pondered whether a contributing factor may be the generation of additional arrhythmogenic potential where tissue is incompletely ablated. This study took place in 2010‐2012 in the absence of Ablation Index (AI) guidance.

Further rhythm correlation and validation of the VHA tool would be useful to add to the growing data.

## CONFLICT OF INTEREST

The authors declare no conflict of interests for this article.
